# Design of Novel Saposin-like Bacteriocins Using a Hybrid Approach

**DOI:** 10.1007/s12602-024-10264-w

**Published:** 2024-05-07

**Authors:** Thomas F. Oftedal, Dzung B. Diep, Morten Kjos

**Affiliations:** Faculty of Chemistry, Biotechnology and Food Science, Norwegian University of Life Sciences, Ås, Norway

**Keywords:** Bacteriocins, Hybrid, Leaderless, Antimicrobial, Pathogens, Inhibition, Killing

## Abstract

A multitude of approaches will be required to respond to the threat posed by the emergence and spread of antibiotic resistant pathogens. Bacteriocins have gained increasing attention as a possible alternative to antibiotics, as such peptide antimicrobials have mechanisms of action different from antibiotics and are therefore equally potent against antibiotic resistant bacteria as their susceptible counterparts. A group of bacteriocins known as saposin-like bacteriocins is believed to act directly on the bacterial membrane. Based on seven saposin-like leaderless bacteriocins, we have constructed a library of hybrid peptides containing all combinations of the N- and C-terminal halves of the native bacteriocins. All hybrid peptides were synthesized using in vitro protein expression and assayed for antimicrobial activity towards several pathogens. Of the 42 hybrid peptides, antimicrobial activity was confirmed for 11 novel hybrid peptides. Furthermore, several of the hybrid peptides exhibited altered antimicrobial spectra and apparent increase in potency compared to the peptides from which they were derived. The most promising hybrid, termed ISP26, was then obtained synthetically and shown to inhibit most of the Gram-positive species tested, including opportunistic pathogens and food spoilage bacteria. Additionally, ISP26 was shown to inhibit *Acinetobacter*, a species of Gram-negative bacteria frequently isolated from nosocomial infections. The activity of the hybrid library provides valuable insights into the design and screening of new active bacteriocins.

**Supplementary Information:**

The online version contains supplementary material available at 10.1007/s12602-024-10264-w.

## Introduction

Antimicrobial resistance (AMR) among bacteria causing infections in humans is increasing. Fewer treatment options are available for infections caused by resistant bacteria, leading to increased morbidity and mortality. An estimated 1.27 million deaths were directly attributable to bacterial antimicrobial resistance (AMR) in 2019 [1]. A review on AMR published in 2016 estimated that an additional 10 million deaths will be caused by AMR in 2050 if current trends continue [2]. Furthermore, the rise of AMR is hastened by viral outbreaks such as the COVID-19 pandemic; from 2019 to 2020, an increase in infections caused by resistant bacteria such as carbapenem-resistant *Acinetobacter* (78% increase), vancomycin-resistant *Enterococcus* (14%), and methicillin-resistant *Staphylococcus aureus* (13%) was reported [3]. The antibiotic resistance crisis is believed to be exacerbated by excessive and inappropriate use of antibiotics in human medicine and agriculture [4]. For this reason, alternative antimicrobials are sorely needed. Peptides and proteins with antimicrobial activity are produced by virtually all organisms as part of their innate immune system. Bacteria also produce antimicrobial peptides and proteins, known as bacteriocins, to inhibit each other during competition for common nutrients or niches [5–9]. Bacteriocins are characterized by a narrow spectrum of activity, often only inhibiting strains closely related to the producer. Furthermore, they often exhibit very potent activity in the pico- to nanomolar range towards their target strains and are thus in some cases considerably more potent than antibiotics [10]. Indeed, bacteriocins have received increasing attention as an alternative or supplement to antibiotics [11, 12].

Bacteriocins are very diverse, differing in sizes, structures, modes of action, molecular targets, and spectrum of activity. Currently, small bacteriocins (< 10 kDa) are typically classified as class I if they are post-translationally modified and class II if they are unmodified [13]. The peptides are further subdivided in both classes based on similarities in biosynthesis, structure, or sequence. Class I is the most diverse group and contains at least 12 subclasses, each based on their characteristic post-translational modification. Examples include the lantibiotics which contain lanthionine; glycocins, which are glycosylated; and circular bacteriocins, where the N- and C-termini are linked. The class II bacteriocins are generally only categorized into four subclasses (IIa–d): IIa, pediocin-like; IIb, two-peptide; IIc, leaderless; and IId, linear non-pediocin-like peptides [13]. Most bacteriocins in both class I and class II are synthesized as precursor peptides with a leader sequence that is removed during or following export to yield the active bacteriocin [13, 14]. A notable exception is the class IIc leaderless bacteriocins, which are unmodified peptides synthesized in the cell in their active form [15].

To date, class IIc includes over 20 bacteriocins which are either single-, two-, or multi-peptide bacteriocins. Among the single-peptide leaderless bacteriocins are the AurA53-like, LsbB-like, and EntL50-like groups of peptides [15, 16]. The LsbB-like bacteriocins depends on the presence of a zinc metalloprotease (RseP/Eep/YvjB) for its antimicrobial activity and kills target cells via a specific interaction with the metalloprotease [17, 18]. In contrast, all non-LsbB leaderless bacteriocins is generally believed to act directly on the bacterial membrane leading to perturbation and permeation without requiring any specific protein on the cell surface as a receptor [15]. A common feature of the non-LsbB leaderless bacteriocins is that they all seem to have a saposin-like fold [19]. In fact, it has been suggested to group the leaderless bacteriocins in two classes based on this difference in structure, namely the saposin-like and the LsbB-like [20]. In addition to the leaderless saposin-like bacteriocins, many circular bacteriocins from class I have the similar saposin-like fold, such as enterocin AS-48 (AS-48) [19].

Saposins are four (A–D) small (~9 kDa) heat-stable proteins with an important function in sphingolipid metabolism in vertebrates [21, 22]. The four saposins are produced in the lysosome or late endosome by proteolytic processing of a single 70-kDa precursor protein prosaposin [20]. Although the four saposins share relatively little sequence identity (20–40%), all four share a very similar compact globular fold consisting of four amphipathic α-helices [23–25]. Acidification of saposins promotes a change in conformation to an open lipid-binding form, either as a dimer or higher oligomer [23, 26–31]. The open conformation exposes a highly hydrophobic core capable of binding the alkyl chain of certain lipids, thus facilitating lipid extraction and solubilization from the membrane [32, 33]. In saposin C, the protonation of several acidic residues is thought to trigger the conformational change to a lipid-binding state [34, 35]. Despite the structural and functional similarities between the four saposins, they differ in their lipid specificities [36]. It is not known how the “bi-functional” property of the saposin-fold in both lipid recognition and conformational switching is achieved. The bacteriocin enterocin AS-48 binds to lipids and is similarly thought to dimerize in a pH-dependent manner [37].

As the arsenal of known bacteriocins is growing, discovering new bacteriocin peptides becomes increasingly difficult. The process of sampling, screening, purifying, and identifying bacteriocins is laborious and time-consuming, and often results with the identification of already described bacteriocins. The discovery of new suitable bacteriocins is arguably one of the bottlenecks in developing these peptides for biomedical applications. Recent advances in synthetic DNA combined with in vitro protein expression enable the direct synthesis of active bacteriocins [38]. In this work, we use a hybrid approach to show that existing bacteriocin families constitute a rich source of new synthetic antimicrobial peptides.

## Results

### Design of Hybrid Bacteriocins

**Fig. 1:**
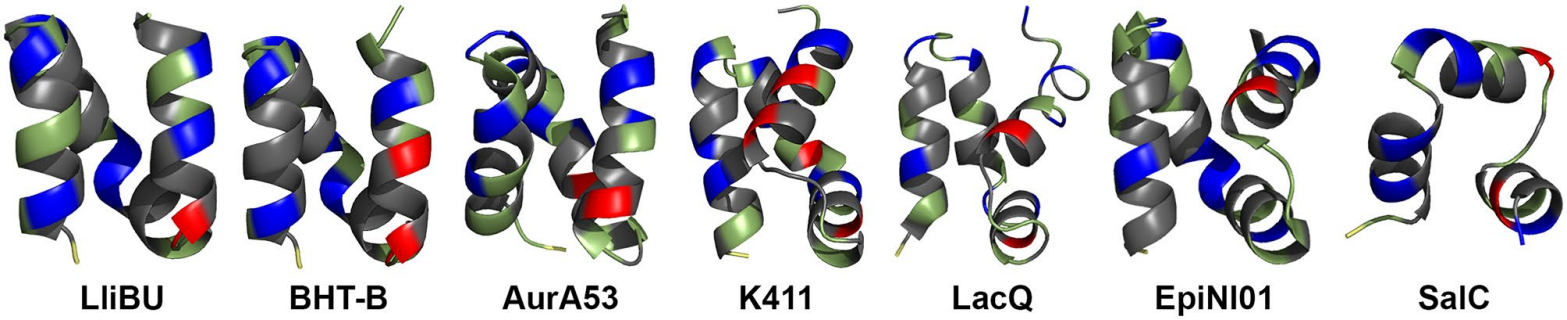
Structures of the leaderless bacteriocins (from left to right): LliBU, BHT-B, AurA53 (PDB ID: 2N8O), K411, LacQ (PDB ID: 2N8P), EpiNI01 (PDB ID: 6SIG), and SalC. Structures with a PDB identifier (ID) have been solved experimentally while the remaining structures were predicted by AlphaFold2. Basic amino acids (K, L, H) are colored blue, acidic residues (D, E) are shown in red, and hydrophobic residues (F, I, L, M, V, W, A, P) are colored in gray. The N-terminal methionine is indicated in yellow

The saposin-like bacteriocins are remarkably similar in structure, but differ in their activity and spectrum, indicating differences in their mechanism despite the structural similarity [39]. A mechanism has been suggested for the circular bacteriocin AS-48 which has a similar saposin-like fold, where protonation of acidic residues is thought to occur at the membrane interface that facilitates membrane insertion [37]. Interestingly, many non-LsbB leaderless bacteriocins also contain one or more acidic residues, primarily located at the C-terminal half of the peptides. As such, we hypothesized that these bacteriocins have a bi-functional property where the C-terminal half is involved in binding to specific lipids unique to sensitive cells while the N-terminal half inserts into the membrane. Based on this assumption, we constructed a library of genes encoding peptide sequences designated ISP1 (ISP, in vitro synthesized peptide) through ISP49 containing all combinations of the N- and C-terminal halves of seven leaderless bacteriocins; lactolisterin BU (LliBU; ISP1), mutacin BHT-B (BHT-B; ISP8), aureocin A53 (AurA53; ISP15), K411 (ISP22), lacticin Q (LacQ; ISP29), epidermicin NI01 (EpiNI01; ISP36), and salivaricin C (SalC; ISP43) (Fig. 1; Online Resource Supplementary Material Table S1).

The resulting library (Table 1) contains 49 peptide sequences, of which 42 are novel hybrid peptides. Despite exchanging the N- and C-terminal halves between the bacteriocins, structure predictions of the hybrids suggest that the saposin-like fold is not considerably affected (Online Resource Supplementary Fig. S1). The genes encoding the seven peptide sequences from which the hybrids were derived were included as a comparison and control.

**Table 1:**
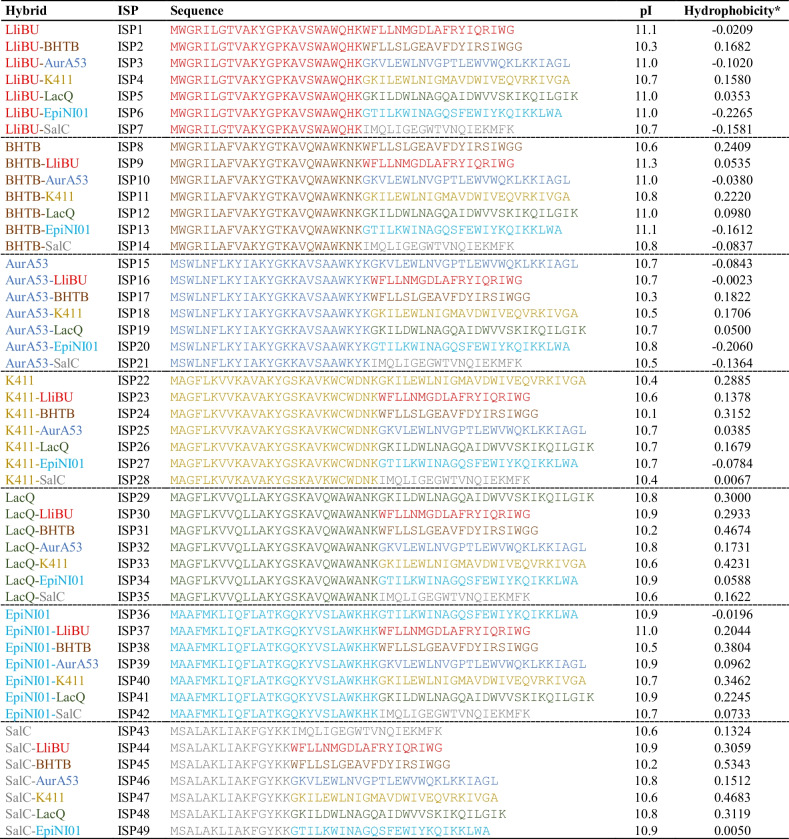
Overview of the peptide sequences encoded by the library (ISP; in vitro synthesized peptide). The isoelectric point (pI) and hydrophobicity were calculated using the Peptides (v2.4.4) library for R v4.1.1 [40]

*Kyte-Doolittle scale

### In Vitro Synthesis and Antimicrobial Activity Screening of Hybrid Bacteriocins

**Fig. 2:**
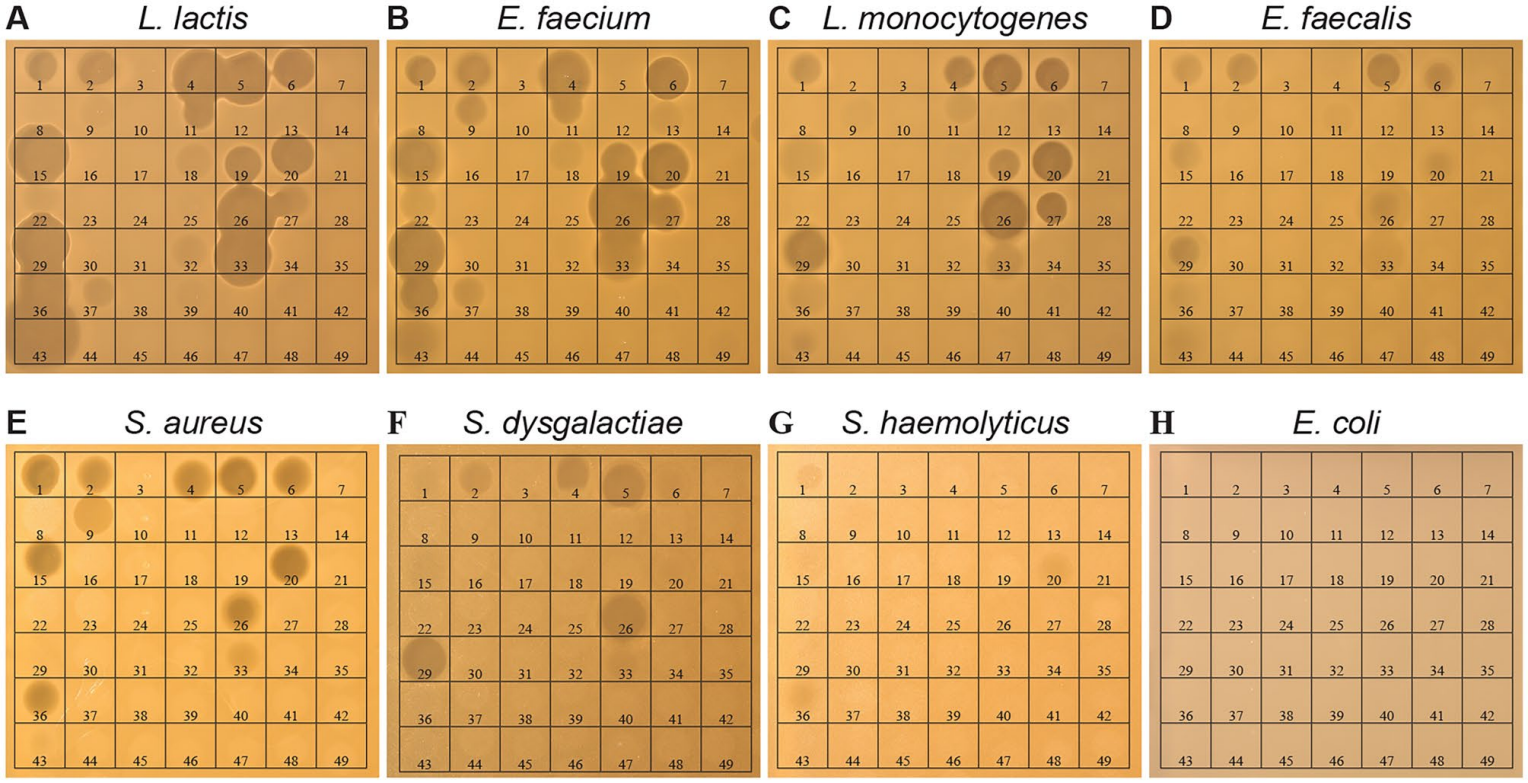
Spot-on-lawn assay assessing antimicrobial activity of the seven leaderless bacteriocins (first column) and hybrid peptides synthesized in vitro. Reaction mixtures of each peptide (ISP1-ISP49, corresponding to Table 1) were spotted inside the corresponding squares (5 µl). Indicators used were **A** *Lac. lactis* subsp. *lactis* IL1403, **B** *Ent. faecium* LMGT 3104, **C** *L. monocytogenes* LMGT 2653, **D** *Ent. faecalis* LMGT 2333, **E** *Staph. aureus* ATCC 14458, **F** *Strep. dysgalactiae* LMGT 3890, **G*** Staph. haemolyticus* LMGT 4071, and **H** *E. coli* DH5α

Genes encoding all the peptides in the library, including the native peptides, were used as a template for in vitro protein expression using PURExpress^®^ In Vitro Protein Synthesis Kit (New England Biolabs). The resulting products from the in vitro synthesis were directly assayed for antimicrobial activity. Using a spot-on-lawn assay, each reaction was tested for antimicrobial activity against a panel of eight indicator bacteria: *Enterococcus faecium*, *Enterococcus faecalis*, *Listeria monocytogenes*, *Staphylococcus aureus*, *Streptococcus dysgalactiae*, *Staphylococcus haemolyticus*, *Lactococcus lactis* subsp. *lactis*, and *Escherichia coli* (Fig. 2).

Of the seven native bacteriocins tested, six of them displayed activity against at least one indicator. From the hybrid peptides tested, 11 (11/42; 26%) of them showed activity against at least one indicator (Table 2). The hybrids ISP4, ISP5, ISP6, ISP20, and ISP26 produced large zones of inhibition against several of the pathogenic strains. Particularly active was ISP26 which inhibited all strains except for *S. haemolyticus* and *E. coli*. ISP4 displayed activity against *Lac. lactis*, *E. faecium*, *L. monocytogenes*, and *S. aureus*, but was inactive against *E. faecalis*. Similarly, ISP5 was inactive against *E. faecium* but displayed good activity against the other strains, except for *S. haemolyticus* and *E. coli*.

**Table 2:**
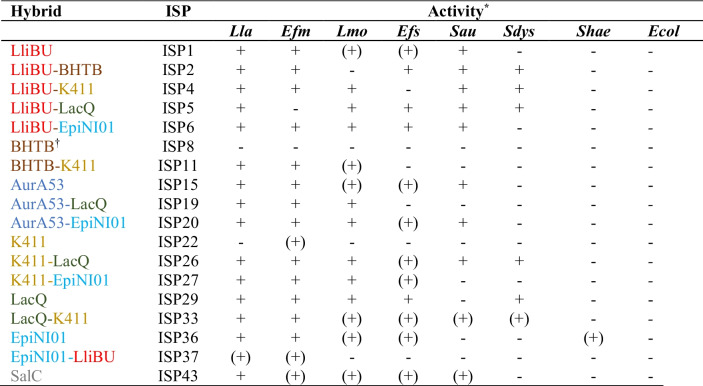
Overview of bacteriocins and hybrid peptides exhibiting inhibition of at least one of the indicators. Clear zone of inhibition, +; diffuse zone of inhibition, (+); no inhibition, -

^*^Lla *Lac. lactis* subsp. *lactis*, Efm *Ent. faecium*, Lmo *L. monocytogenes*, Efs *Ent. faecalis*, Sau *Staph. aureus*, Sdys *Strep. dysgalactiae*, Shae *Staph. haemolyticus*, Ecol *E. coli*

^†^Synthesis of this peptide likely failed or had very low yield (see the “Discussion” section)

It was also interesting to note that some of the hybrid peptides appeared to display altered inhibition spectra compared to their native counterparts. AurA53 and EpiNI01 showed only weak activity against *L. monocytogenes*, while AurA53-EpiNI01 (ISP20) showed good activity (clear zone of inhibition). Similarly, LliBU and K411 had no or negligible effect against *Strep. dysgalactiae*; an inhibition zone was observed for the LliBU-K411. In fact, four of the hybrids with the N-terminal half from LliBU (4/7; ~ 60%) showed good activity towards several indicators, while only one hybrid with the C-terminal half from LliBU (ISP37) showed weak activity.

To further assess the antimicrobial activity of these hybrid bacteriocins, we sought to establish a bacterial production scheme that would allow us to obtain larger quantities of the hybrid peptides. However, obtaining active peptides by heterologous expression was not successful (see Online Resource Supplementary Material). The most promising hybrid, ISP26 (K411-LacQ), was therefore obtained synthetically which allowed us to confirm its antimicrobial activity and to assay its activity against a larger panel of bacterial species (Table 3).

**Table 3:** Antimicrobial activity of synthetic ISP26 against a larger panel of bacterial species. Using a spot-on-lawn assay, 5 µl of ISP26 was spotted on plates overlaid with the indicator. Pure DMSO was also spotted in equal volume as a negative control and showed no clear zones of inhibition against any of the indicators. A clear zone of inhibition at 0.01 mg/ml, +++; 0.1 mg/ml, ++; 1 mg/ml, +; no inhibition, -

Strain*	**ISP26 activity**
*Bacillus cereus* LMGT 2805	++
*B. cereus* ATCC 9139 B	++
*B. cereus* LMGT 2731	++
*Staph. haemolyticus* LMGT 4133	++
*Staph. simulans* LMGT 3233	-
*Staph. arlettae* LMGT 4134	+
*Staph. hominis* LMGT 3129	++
*Staph. epidermidis* LMGT 3522	-
*Staph. aureus* LMGT 3022	++
*Staph. aureus* ATCC 14458	+
*Staph. aureus* LMGT 3242	-
*Carnobacterium divergens* NCDO 2306	+++
*Carno. piscicola* LMGT 3465	+++
*Ent. avium* LMGT 3465	++
*Ent. faecalis* LMGT 2333	+
*Ent. faecalis* LMGT 3331	++
*Ent. faecalis* LMGT 3332	+
*Ent. faecium* LMGT 2763	++
*Ent. faecium* LMGT 2772	+++
*Ent. faecium* LMGT 2783	++
*Lact. curvatus* LMGT 2353	++
*Lact. curvatus* LMGT 2355	++
*Lact. plantarum* LMGT 2003	+++
*Lact. plantarum* LMGT 2352	++
*Lact. plantarum* LMGT 3125	++
*Lact. sakei* LMGT 2361	+++
*Lact. sakei* LMGT 2380	+++
*Lact. salivarius* LMGT 2787	+
*Lac. garvieae* LMGT 3390	++
*Lac. lactis* IL1403	++
*Lac. lactis* LMGT 2081	+++
*Leuconostoc gelidum* LMGT 2386	+
*Listeria innocua* LMGT 2710	++
*L. innocua* LMGT 2785	+++
*L. ivanovii* LMGT 2813	++
*L. monocytogenes* LMGT 2604	+
*L. monocytogenes* LMGT 2650	++
*L. monocytogenes* LMGT 2651	+++
*Pediococcus acidilactici* LMGT 2002	+++
*Ped. pentosaceus* LMGT 2001	++
*Ped. pentosaceus* LMGT 2366	+
*Strep. agalactiae* LMGT 3347	++
*Strep. dysgalactiae* LMGT 3890	++
*Strep. dysgalactiae* LMGT 3899	++
*Strep. thermophilus* SFi13	+++
*Strep. salivarius* LMGT 3597	++
*Strep. uberis* LMGT 3912	+
*Strep. uberis* LMGT 3918	++
*Trueperella pyogenes* LMGT 3952	++
*E. coli* DH5α	-
*E. coli* TG1	-
*Salmonella typhimurium* B1377	-
*Salm. enteritidis* B1378	-
*Serratia* sp. B933	-
*Serratia* sp. B934	-
*Pseudomonas aeruginosa* LMGT 3294	-
*Acinetobacter baumannii* B1384	+
*Candida albicans* B1400	-

*LMGT/B, Laboratory of Microbial Gene Technology (LMGT), Norwegian University of Life Sciences, Ås, Norway

Using a spot-on-lawn antimicrobial assay, the synthetically obtained ISP26 was shown to have broad-spectrum activity against most Gram-positive species tested, including *Bacillus*, *Lactobacillus*, *Lactococcus*, *Listeria*, *Pediococcus*, and *Streptococcus*. The exception is staphylococci, where sensitivity towards ISP26 varied considerably between species and strains. For example, while activity is observed against *Staph. haemolyticus* and *Staph. hominis*, *Staph. epidermidis* appears insensitive to ISP26. Furthermore, among the *S. aureus* strains tested initially, *S. aureus* LMGT 3022 showed a clear zone of inhibition at 0.1 mg/ml while *S. aureus* LMGT 3242 was not inhibited even at 1 mg/ml of the bacteriocin. To investigate if the variation in sensitivity was peculiar to the tested strains, ISP26 was further assayed for activity against a panel of 134 *S. aureus* strains from our laboratory collection (Online Resource Table S3).

Of all tested strains, 77 (58%) were insensitive to ISP26. Interestingly, while most of the Gram-negative species tested were insensitive to ISP26, *Acinetobacter baumannii* was shown to be sensitive to ISP26. To further explore the activity of ISP26 against *Acinetobacter* sp., another panel of indicators belonging to this genus was assayed for sensitivity (Table 4; Online Resource Supplemental Material Fig. S1).

**Table 4:** Antimicrobial activity of ISP26 against some *Acinetobacter* species. Clear zone of inhibition at 0.01 mg/ml, +++; 0.1 mg/ml, ++; 1 mg/ml, +; no inhibition, -

Strain*	**ISP26 activity**
*Ac. pittii* B1379	+
*Ac. ursingii* B1391	++
*Ac. soli* B1392	+
*Ac. radioresistens* B1393	+
*Ac. radioresistens* B1394	+
*Ac. towneri* B1395	++
*Ac. calcoaceticus* B1396	+
*Ac. lwoffii* B1397	++
*Ac.* gen. sp. 9	++

*LMGT/B, Laboratory of Microbial Gene Technology (LMGT), Norwegian University of Life Sciences, Ås, Norway

All species of *Acinetobacter* that was tested showed sensitivity towards bacteriocin ISP26 at 1 mg/ml and four species were also inhibited at 0.1 mg/ml (*Ac. ursingii*, *Ac. towneri*, *Ac. lwoffii*, *Ac.* gen sp. 9), clearly demonstrating the antimicrobial effect of ISP26 against *Acinetobacter*.

## Discussion

The construction of the hybrid bacteriocins presented in this work revealed active new-to-nature bacteriocins with increased inhibition spectrum and an apparent increase in potency compared to their native counterparts. The constructed hybrid bacteriocins were shown to inhibit the growth of WHO priority pathogens, including *Ac. baumannii, Staph. aureus* and *Ent. faecium*, and other important pathogens such as *Ent. faecalis* and *L. monocytogenes*. As such, these hybrid peptides may serve as an important addition or supplement to antibiotics for the treatment of infections from these pathogens in the future.

Notably, the hybrid peptide ISP26 displayed broad spectrum antimicrobial activity, including also Gram-negative *Ac. baumannii* and other *Acinetobacter* species. Antimicrobial activities of bacteriocins derived from Gram-positive bacteria active against Gram-negative species are quite rare, and only a few examples of bacteriocins with activity against *Ac. baumannii* have been reported [41, 42]. Multidrug- or even pandrug-resistant *Ac. baumannii* are emerging pathogens associated with a range of infections, and there is a pressing need to find new therapeutics [43, 44]. It should therefore be further explored whether saposin-like bacteriocins could be used alone or in combination with other antimicrobials to fight *Ac. baumannii* infections.

To design the library of genes encoding hybrid bacteriocins in this work, we hypothesized that the saposin-like bacteriocins are “bifunctional,” where the N- and C-terminal halves of the peptides serve different functions. This idea is derived from a proposed mechanism of action for the circular bacteriocin AS-48, and structure and sequence similarities shared between this bacteriocin and other saposin-like bacteriocin [19, 37, 39]. In the proposed mechanism for AS-48, protonation of four glutamic acid side chains is believed to occur in the acidic environment of the membrane interface, resulting in the transition to a membrane-interacting dimeric form. The protonated glutamic acids of AS-48 recognize and associate with the phosphate moiety of a phospholipid, which was demonstrated by crystallography [37]. It is not known whether the non-LsbB leaderless bacteriocins act as single peptides or form dimers or multimers, however, many saposin-like peptides (SAPLIPs) are known to form dimers in their active form [31, 45, 46]. In fact, the transition to the dimeric form for many SAPLIPs is believed to occur upon interacting with the membrane [24, 25, 31, 47]. If a similar mechanism is also employed by the saposin-like bacteriocins, their activity and inhibition spectrum may depend on the presence of, or abundance of, certain phospholipids that will vary between species. However, the strain-to-strain differences in sensitivity of *S. aureus* against ISP26 may suggest the involvement of specific cell surface features (Online Resource Table S3). Although our hybrid approach successfully resulted in saposin-like hybrid peptides with improved activities, we could not assign any obvious or distinct role of the N- and C-terminal halves of the peptides. Understanding the mechanism and differences in activity between the hybrids will thus need more investigation.

Our data shows that in vitro protein synthesis is a well-suited tool to screen for active, new-to-nature antimicrobials. Nevertheless, there are numerous challenges with in vitro synthesis of bacteriocin-like peptides, which practically limit this method to small-scale screening. For example, we have experienced that antimicrobial activity from reactions is lost upon storage/incubation past 24 h, presumably due to aggregation and/or precipitation [48]. We have also not been successful at purifying the peptides from the reaction mixture. Furthermore, in vitro synthesis of bacteriocins sometimes fails, which was also evident in our study. For example, the native bacteriocin BHT-B was included as a control in the screen and known to be active towards *Lac. lactis* [49]. However, no inhibition zone was produced by the in vitro expression of BHT-B in our screen, indicating low- or no yield from the reaction. It is therefore likely that more of the hybrid peptides with no reported activity in the screen (Fig. 1) may be false negatives due to the failed synthesis of some peptides. The variation in synthesis efficiency also precludes the direct comparison of potency between the peptides. Our results suggest that several of the hybrid peptides have higher potency than the peptides from which they were derived (e.g., ISP4-6 has larger zone than ISP1 towards *Lac. lactis*, see Fig. 1); however, we cannot exclude that this is just a result of different synthesis efficiency in the in vitro reactions.

Due to the length (50–53 aa) and high hydrophobicity of the peptides, commercial peptide synthesis can be difficult and costly. For this reason, we sought to produce the hybrid bacteriocins heterologously in *E. coli,* the only species insensitive to all peptides tested. However, all our attempts at expressing and purifying these hybrid peptides in *E. coli* were unsuccessful (see Online Resource Supplementary Material). The expression of ISP26 and ISP29 in *E. coli* as C-terminal fusions to maltose-binding protein (MBP) also failed. We could not determine why expression failed in *E. coli*, although clones expressing MBP-ISP26/29 exhibited severely attenuated growth. It could be speculated that these peptides are active when delivered in the cytoplasm of *E. coli* (e.g., inner membrane perturbation/permeabilization), and that the lack of activity in our screen is due to the outer membrane barrier. A failure to express a similar protein fusion in *E. coli* was recently reported by Malesevic et al.; in this work, the authors tried to express a fusion of LliBU (ISP1) to MBP [50]. LliBU is a peptide of similar physicochemical properties as the hybrid peptides. It has also been documented that *E. coli* has a great ability to degrade peptides, such as bacteriocins, when fused to proteins [51–53].

Furthermore, since the native producers of all bacteriocins in the library are Gram-positive species, nisin-inducible expression of the hybrid peptide in the Gram-positive *Lac. lactis* was attempted (see Online Resource Supplementary Material). While we were able to heterologously express and purify MBP-ISP26 in *Lac. lactis* NZ9000 (Online Resource Supplemental Material Fig. S2), the release of functional (antimicrobial) ISP26 from the fusion protein using the incorporated TEV cleavage tag was unsuccessful with the set of conditions tested here. Thus, alternative methods and design of the constructs to express and purify the peptide may be needed to overcome this problem in the future. This could, for example, include so-called sandwich approaches, where both termini of the peptide are fused to innocuous proteins that can be excised without scars (e.g., SUMO and intein proteins) [51, 52].

In this work, we show that previously characterized leaderless bacteriocins can serve as scaffolds for the construction of new-to-nature antimicrobials with improved properties. Additionally, screening new sequences for antimicrobial activity can provide invaluable insight into antimicrobial determinants. Very little is known about the mechanism of most bacteriocins and the factors that determine their potency and spectrum. A better understanding of the features shared among active peptides versus inactive ones, and those active towards certain species but not others, can allow us to rationally design new peptides targeting high priority pathogens. However, more research is needed to fully characterize these peptides and to assess their therapeutic potential as well as to find cost-effective strategies for their production.

## Materials and Methods

### Strains and Growth Conditions

All strains used in this study are listed in Table 5. *Lac. lactis* was grown at 30 °C in M17 broth (Oxoid) supplemented with 0.4% glucose (GM17), and *E. coli* was grown in LB at 37 °C (180 rpm). All remaining strains were grown in BHI (brain heart infusion; Oxoid) at 37 °C without shaking.

**Table 5:** Indicator strains used for assessing activity and inhibition spectrum of in vitro synthesized peptides

**Indicator strain**	**Reference**
*Lactococcus lactis* IL1403	[54]
*Enterococcus faecium* LMG 20705*	[55]
*Listeria monocytogenes* LMGT 2653*	Lab collection (LMGT), Norway
*Streptococcus dysgalactiae* LMGT 3890*	Lab collection (LMGT), Norway
*Enterococcus faecalis* LMGT 2333	Lab collection (LMGT), Norway
*Staphylococcus aureus* ATCC 14458*	ATCC
*Staphylococcus haemolyticus* LMGT 4071*	[56]
*Escherichia coli* DH5α	Invitrogen (Cat. No. 18265–017)

Laboratory of Microbial Gene Technology (LMGT), Norwegian University of Life Sciences, Ås, Norway

*Clinical isolates

### In Vitro Protein Expression and Antimicrobial Assay

Bacteriocin peptide sequences were reverse translated and codon was optimized for *E. coli* K12 using GENEius (Eurofins Genomics, Germany). All genes were synthesized by Pepmic Co. Ltd (Suzhou, China) and supplied in pET-3a. Plasmids were solubilized to 250 ng/µl in Milli-Q water and used directly as templates for in vitro protein synthesis using PURExpress^®^ In Vitro Protein Synthesis Kit (New England Biolabs). Reactions of 50 µl using 500 ng of template per reaction were assembled according to the protocol provided by the manufacturer in a 96-well plate. The 96-well plate was sealed using heat-sealing film and incubated at 30 °C for 4 h with vigorous shaking at 1200 rpm using a microplate shaker (PMS-1000i, Grant-Bio, Grant Instruments Ltd., Shepreth, UK). All reactions were immediately assayed for antimicrobial activity using a spot-on-lawn assay. Briefly, an overnight culture of the indicator strain was diluted 50-fold in growth medium (see above) containing 0.8% agar and poured over an agar plate (10 × 10 cm, square). After solidification, 5 µl of each reaction mixture was spotted onto the plate and allowed to dry. All plates were incubated at 30 °C overnight for the appearance of inhibition zones.

The peptide ISP26 was obtained from Pepmic Co. Ltd (Suzhou, China) at > 95% purity and solubilized in pure molecular biology grade dimethyl sulfoxide (DMSO; Sigma-Aldrich, D8418) to a stock concentration of 1 mg/ml (170 µM); all dilutions were prepared in DMSO. Sensitivity towards ISP26 was determined using a spot-on-lawn assay as described above, with the exception of *Acinetobacter* which was assayed according to the EUCAST disk diffusion methodology (Online Resource Supplemental Material Fig. S2). For *Acinetobacter* species, colonies were suspended in saline (0.9% NaCl) until McFarland 0.5 and spread on a Mueller–Hinton agar plate using a sterile cotton swab. ISP26 was spotted (5 µl) on the agar plates as indicated. An equal volume of pure DMSO was spotted in all assays as a negative control.

## Supplementary Information

Below is the link to the electronic supplementary material.

Supplementary file1 (DOCX 1325 KB)

## Data Availability

No datasets were generated or analysed during the current study.

## References

[1] Murray CJL, Ikuta KS, Sharara F et al (2022) Global burden of bacterial antimicrobial resistance in 2019: a systematic analysis. Lancet 399:629–655. 10.1016/S0140-6736(21)02724-0 35065702 10.1016/S0140-6736(21)02724-0 PMC8841637

[2] O’Neill J (2014) Review on antimicrobial resistance antimicrobial resistance: tackling a crisis for the health and wealth of nations. Wellcome Trust and the UK Department of Health. https://amr-review.org/sites/default/files/AMR%20Review%20Paper%20-%20Tackling%20a%20crisis%20for%20the%20health%20and%20wealth%20of%20nations_1.pdf. Accessed 3 Jan 2024

[3] National Center for Emerging and Zoonotic Infectious Diseases (U.S.). Division of Healthcare Quality Promotion. Division of Healthcare Quality Promotion. (Ed.). (2022). COVID-19: U.S. impact on antimicrobial resistance, special report 2022 (cdc:119025). https://stacks.cdc.gov/view/cdc/119025

[4] Sugden R, Kelly R, Davies S (2016) Combatting antimicrobial resistance globally. Nat Microbiol 1:1–2. 10.1038/nmicrobiol.2016.187 10.1038/nmicrobiol.2016.187 27670123

[5] Kuhar I, Žgur-Bertok D (1999) Transcription regulation of the colicin K *cka* gene reveals induction of colicin synthesis by differential responses to environmental signals. J Bacteriol 181:7373–7380. 10.1128/jb.181.23.7373-7380.1999 10572143 10.1128/jb.181.23.7373-7380.1999 PMC103702

[6] O’Sullivan JN, Rea MC, O’Connor PM et al (2019) Human skin microbiota is a rich source of bacteriocin-producing staphylococci that kill human pathogens. FEMS Microbiol Ecol 95:fiy241. 10.1093/femsec/fiy241 30590567 10.1093/femsec/fiy241 PMC6340406

[7] Zipperer A, Konnerth MC, Laux C et al (2016) Human commensals producing a novel antibiotic impair pathogen colonization. Nature 535:511–516. 10.1038/nature18634 27466123 10.1038/nature18634

[8] Nakatsuji T, Chen TH, Narala S et al (2017) Antimicrobials from human skin commensal bacteria protect against *Staphylococcus aureus* and are deficient in atopic dermatitis. Sci Transl Med 9:eaah4680. 10.1126/scitranslmed.aah4680 28228596 10.1126/scitranslmed.aah4680 PMC5600545

[9] Sassone-Corsi M, Nuccio S-P, Liu H et al (2016) Microcins mediate competition among Enterobacteriaceae in the inflamed gut. Nature 540:280–283. 10.1038/nature20557 27798599 10.1038/nature20557 PMC5145735

[10] Hassan M, Kjos M, Nes IF et al (2012) Natural antimicrobial peptides from bacteria: characteristics and potential applications to fight against antibiotic resistance. J Appl Microbiol 113:723–736. 10.1111/j.1365-2672.2012.05338.x 22583565 10.1111/j.1365-2672.2012.05338.x

[11] Cotter PD, Ross RP, Hill C (2013) Bacteriocins — a viable alternative to antibiotics? Nat Rev Microbiol 11:95–105. 10.1038/nrmicro2937 23268227 10.1038/nrmicro2937

[12] Sang Y, Blecha F (2008) Antimicrobial peptides and bacteriocins: alternatives to traditional antibiotics. Anim Health Res Rev 9:227–235. 10.1017/S1466252308001497 18983725 10.1017/S1466252308001497

[13] Antoshina DV, Balandin SV, Ovchinnikova TV (2022) Structural features, mechanisms of action, and prospects for practical application of class II bacteriocins. Biochemistry Moscow 87:1387–1403. 10.1134/S0006297922110165 36509729 10.1134/S0006297922110165

[14] Arnison PG, Bibb MJ, Bierbaum G et al (2012) Ribosomally synthesized and post-translationally modified peptide natural products: overview and recommendations for a universal nomenclature. Nat Prod Rep 30:108–160. 10.1039/C2NP20085F 10.1039/c2np20085f PMC3954855 23165928

[15] Perez RH, Zendo T, Sonomoto K (2018) Circular and leaderless bacteriocins: biosynthesis, mode of action, applications, and prospects. Front Microbiol. 10.3389/fmicb.2018.02085 30233551 10.3389/fmicb.2018.02085 PMC6131525

[16] Tymoszewska A, Ovchinnikov KV, Diep DB et al (2021) *Lactococcus lactis* resistance to aureocin A53- and enterocin L50-like bacteriocins and membrane-targeting peptide antibiotics relies on the YsaCB-KinG-LlrG four-component system. Antimicrob Agents Chemother. 10.1128/aac.00921-21 34516250 10.1128/AAC.00921-21 PMC8597747

[17] Ovchinnikov KV, Kristiansen PE, Straume D et al (2017) The leaderless bacteriocin enterocin K1 is highly potent against *Enterococcus faecium*: a study on structure, target spectrum and receptor. Front Microbiol 8:774. 10.3389/fmicb.2017.00774 28515717 10.3389/fmicb.2017.00774 PMC5413573

[18] Uzelac G, Kojic M, Lozo J et al (2013) A Zn-dependent metallopeptidase is responsible for sensitivity to LsbB, a class II leaderless bacteriocin of *Lactococcus lactis* subsp. *lactis* BGMN1-5. J Bacteriol 195:5614–5621. 10.1128/jb.00859-13 24123824 10.1128/JB.00859-13 PMC3889605

[19] Towle KM, Vederas JC (2017) Structural features of many circular and leaderless bacteriocins are similar to those in saposins and saposin-like peptides. Med Chem Commun 8:276–285. 10.1039/C6MD00607H 10.1039/c6md00607h PMC6072434 30108744

[20] Yi Y, Li P, Zhao F et al (2022) Current status and potentiality of class II bacteriocins from lactic acid bacteria: structure, mode of action and applications in the food industry. Trends Food Sci Technol 120:387–401. 10.1016/j.tifs.2022.01.018

[21] Kolter T, Sandhoff K (2010) Lysosomal degradation of membrane lipids. FEBS Lett 584:1700–1712. 10.1016/j.febslet.2009.10.021 19836391 10.1016/j.febslet.2009.10.021

[22] Hazkani-Covo E, Altman N, Horowitz M, Graur D (2002) The evolutionary history of prosaposin: two successive tandem-duplication events gave rise to the four saposin domains in vertebrates. J Mol Evol 54:30–34. 10.1007/s00239-001-0014-0 11734895 10.1007/s00239-001-0014-0

[23] Gebai A, Gorelik A, Nagar B (2018) Crystal structure of saposin D in an open conformation. J Struct Biol 204:145–150. 10.1016/j.jsb.2018.07.011 30026085 10.1016/j.jsb.2018.07.011

[24] Ahn VE, Leyko P, Alattia J-R et al (2006) Crystal structures of saposins A and C. Protein Sci 15:1849–1857. 10.1110/ps.062256606 16823039 10.1110/ps.062256606 PMC2242594

[25] Rossmann M, Schultz-Heienbrok R, Behlke J et al (2008) Crystal structures of human saposins C and D: implications for lipid recognition and membrane interactions. Structure 16:809–817. 10.1016/j.str.2008.02.016 18462685 10.1016/j.str.2008.02.016

[26] Sandin SI, de Alba E (2022) Quantitative studies on the interaction between saposin-like proteins and synthetic lipid membranes. Methods protoc 5:19. 10.3390/mps5010019 35200535 10.3390/mps5010019 PMC8878781

[27] Shamin M, Spratley SJ, Graham SC, Deane JE (2021) A tetrameric assembly of saposin A: increasing structural diversity in lipid transfer proteins. Contact. 10.1177/25152564211052382 37143956 10.1177/25152564211052382 PMC7614494

[28] Popovic K, Holyoake J, Pomès R, Privé GG (2012) Structure of saposin A lipoprotein discs. Proc Natl Acad Sci USA 109:2908–2912. 10.1073/pnas.1115743109 22308394 10.1073/pnas.1115743109 PMC3286916

[29] Ciaffoni F, Tatti M, Salvioli R, Vaccaro AM (2003) Interaction of saposin D with membranes: effect of anionic phospholipids and sphingolipids. Biochem J 373:785–792. 10.1042/bj20030359 12733985 10.1042/BJ20030359 PMC1223540

[30] Vaccaro AM, Ciaffoni F, Tatti M et al (1995) pH-dependent conformational properties of saposins and their interactions with phospholipid membranes (∗). J Biol Chem 270:30576–30580. 10.1074/jbc.270.51.30576 8530492 10.1074/jbc.270.51.30576

[31] Ahn VE, Faull KF, Whitelegge JP et al (2003) Crystal structure of saposin B reveals a dimeric shell for lipid binding. Proc Natl Acad Sci USA 100:38–43. 10.1073/pnas.0136947100 12518053 10.1073/pnas.0136947100 PMC140876

[32] León L, Tatituri RVV, Grenha R et al (2012) Saposins utilize two strategies for lipid transfer and CD1 antigen presentation. Proc Natl Acad Sci USA 109:4357–4364. 10.1073/pnas.1200764109 22331868 10.1073/pnas.1200764109 PMC3311357

[33] Alattia J-R, Shaw JE, Yip CM, Privé GG (2006) Direct visualization of saposin remodelling of lipid bilayers. J Mol Biol 362:943–953. 10.1016/j.jmb.2006.08.009 16949605 10.1016/j.jmb.2006.08.009

[34] Abu-Baker S, Qi X, Lorigan GA (2007) Investigating the interaction of saposin C with POPS and POPC phospholipids: a solid-state NMR spectroscopic study. Biophys J 93:3480–3490. 10.1529/biophysj.107.107789 17704143 10.1529/biophysj.107.107789 PMC2072076

[35] de Alba E, Weiler S, Tjandra N (2003) Solution structure of human saposin C: pH-dependent interaction with phospholipid vesicles. Biochemistry 42:14729–14740. 10.1021/bi0301338 14674747 10.1021/bi0301338

[36] Yuan W, Qi X, Tsang P et al (2007) Saposin B is the dominant saposin that facilitates lipid binding to human CD1d molecules. Proc Natl Acad Sci 104:5551–5556. 10.1073/pnas.0700617104 17372201 10.1073/pnas.0700617104 PMC1838443

[37] Sánchez-Barrena MJ, Martínez-Ripoll M, Gálvez A, et al (2003) Structure of bacteriocin AS-48: from soluble state to membrane bound state. J Mol Biol 334:541–549. 10.1016/j.jmb.2003.09.060 14623193 10.1016/j.jmb.2003.09.060

[38] Gabant P, Borrero J (2019) PARAGEN 1.0: a standardized synthetic gene library for fast cell-free bacteriocin synthesis. Front Bioeng Biotechnol. 10.3389/fbioe.2019.00213 31552239 10.3389/fbioe.2019.00213 PMC6743375

[39] Acedo JZ, van Belkum MJ, Lohans CT et al (2016) Nuclear magnetic resonance solution structures of lacticin Q and aureocin A53 reveal a structural motif conserved among leaderless bacteriocins with broad-spectrum activity. Biochemistry 55:733–742. 10.1021/acs.biochem.5b01306 26771761 10.1021/acs.biochem.5b01306

[40] Osorio D, Rondón-Villarreal P, Torres R (2015) Peptides: a package for data mining of antimicrobial peptides. R J 7:4–14. 10.32614/RJ-2015-001

[41] Chi H, Holo H (2018) Synergistic antimicrobial activity between the broad spectrum bacteriocin garvicin KS and nisin, farnesol and polymyxin B against gram-positive and gram-negative bacteria. Curr Microbiol 75:272–277. 10.1007/s00284-017-1375-y 29058043 10.1007/s00284-017-1375-y PMC5809525

[42] Martínez-Trejo A, Ruiz-Ruiz JM, Gonzalez-Avila LU et al (2022) Evasion of antimicrobial activity in *Acinetobacter baumannii* by target site modifications: an effective resistance mechanism. Int J Mol Sci 23:6582. 10.3390/ijms23126582 35743027 10.3390/ijms23126582 PMC9223528

[43] Gallagher P, Baker S (2020) Developing new therapeutic approaches for treating infections caused by multi-drug resistant *Acinetobacter baumannii*: *Acinetobacter baumannii* therapeutics. J Infect 81:857–861. 10.1016/j.jinf.2020.10.016 33115656 10.1016/j.jinf.2020.10.016

[44] Michalopoulos A, Falagas ME (2010) Treatment of *Acinetobacter* infections. Expert Opin Pharmacother 11:779–788. 10.1517/14656561003596350 20210684 10.1517/14656561003596350

[45] Bruhn H (2005) A short guided tour through functional and structural features of saposin-like proteins. Biochem J 389:249–257. 10.1042/BJ20050051 15992358 10.1042/BJ20050051 PMC1175101

[46] Hawkins CA, de Alba E, Tjandra N (2005) Solution structure of human saposin C in a detergent environment. J Mol Biol 346:1381–1392. 10.1016/j.jmb.2004.12.045 15713488 10.1016/j.jmb.2004.12.045

[47] Michalek M, Leippe M (2015) Mechanistic insights into the lipid interaction of an ancient saposin-like protein. Biochemistry 54:1778–1786. 10.1021/acs.biochem.5b00094 25715682 10.1021/acs.biochem.5b00094

[48] Oftedal TF, Ovchinnikov KV, Hestad KA et al (2021) Ubericin K, a new pore-forming bacteriocin targeting mannose-PTS. Microbiol Spectr 9:e00299-e321. 10.1128/Spectrum.00299-21 34643411 10.1128/Spectrum.00299-21 PMC8515946

[49] Hyink O, Balakrishnan M, Tagg JR (2005) *Streptococcus rattus* strain BHT produces both a class I two-component lantibiotic and a class II bacteriocin. FEMS Microbiol Lett 252:235–241. 10.1016/j.femsle.2005.09.003 16194596 10.1016/j.femsle.2005.09.003

[50] Malesevic M, Gardijan L, Miljkovic M et al (2023) Exploring the antibacterial potential of *Lactococcus lactis* subsp. *lactis* bv. diacetylactis BGBU1–4 by genome mining, bacteriocin gene overexpression, and chemical protein synthesis of lactolisterin BU variants. Lett Appl Microbiol 76:ovad004. 10.1093/lambio/ovad004 36695436 10.1093/lambio/ovad004

[51] Lamer T, van Belkum MJ, Wijewardane A et al (2022) SPI “sandwich”: combined SUMO- peptide-intein expression system and isolation procedure for improved stability and yield of peptides. Protein Sci 31:e4316. 10.1002/pro.4316 35481634 10.1002/pro.4316 PMC9045064

[52] Lamer T, Vederas JC (2023) Simplified cloning and isolation of peptides from “sandwiched” SUMO-peptide-intein fusion proteins. BMC Biotechnol 23:11. 10.1186/s12896-023-00779-5 37020212 10.1186/s12896-023-00779-5 PMC10074672

[53] van Belkum MJ, Aleksandrzak-Piekarczyk T, Lamer T, Vederas JC (2023) *Lactococcus lactis* mutants resistant to lactococcin A and garvicin Q reveal missense mutations in the sugar transport domain of the mannose phosphotransferase system. Microbiol Spectr 0:e03130–23. 10.1128/spectrum.03130-23 10.1128/spectrum.03130-23 PMC10783117 38047704

[54] Chopin A, Chopin M-C, Moillo-Batt A, Langella P (1984) Two plasmid-determined restriction and modification systems in *Streptococcus lactis*. Plasmid 11:260–263. 10.1016/0147-619X(84)90033-7 6087394 10.1016/0147-619x(84)90033-7

[55] Rosenbergová Z, Oftedal TF, Ovchinnikov KV et al (2022) Identification of a novel two-peptide lantibiotic from *Vagococcus fluvialis*. Microbiol Spectr 10:e00954–e1022. 10.1128/spectrum.00954-22 35730941 10.1128/spectrum.00954-22 PMC9431498

[56] Kranjec C, Kristensen SS, Bartkiewicz KT et al (2021) A bacteriocin-based treatment option for *Staphylococcus haemolyticus* biofilms. Sci Rep 11:13909. 10.1038/s41598-021-93158-z 34230527 10.1038/s41598-021-93158-z PMC8260761

