# Supplementary material for: Design of Novel Saposin-like Bacteriocins Using a Hybrid Approach
Source: Probiotics Antimicrob Proteins. 2024 May 7;17(5):3156–65. doi: 10.1007/s12602-024-10264-w (PMC12532754; doi:10.1007/s12602-024-10264-w)
Supplement: Supplementary file 1 — Supplementary file1 (DOCX 1325 KB) [file 12602_2024_10264_MOESM1_ESM.docx]

**Supplementary Material**

**Journal:** Probiotics and Antimicrobial Proteins

**Design of Novel Saposin-like Bacteriocins Using a Hybrid Approach**

**Thomas F. Oftedal^1*^, Dzung B. Diep^1†^, Morten Kjos^1^**

^1^ Faculty of Chemistry, Biotechnology and Food Science, Norwegian University of Life Sciences, Ås, Norway

^†^ Died December 7, 2022.

*** Correspondence:**

Thomas F. Oftedal

thof@nmbu.no

**Supplementary Methods**

**Cloning**

The gene encoding ISP26 was amplified from pET-3a using the primers HiFi_ISP26_F and HiFI_ISP26_R (Table S2). The plasmid pMAL-c6T (0.5 µg) was digested for 1 hour at 37°C with SbfI-HF and AlwNI (New England BioLabs). Both were gel-purified using NucleoSpin Gel and PCR Clean-up (Macherey-Nagel), then combined in a molar ratio of 1:10 (vector:insert) and assembled using a GeneArt ™ Gibson Assembly® HiFi master mix (Thermo Fisher Scientific) according to the manufacturer’s instructions. The Gibson assembly mixture was used to transform *E. coli* DH5α. The plasmid was isolated using EZNA Plasmid DNA Mini Kit I (Omega Bio-Tek). The gene fusion was amplified from the plasmid (or from pMAL-c6T) using the primers ISP_BamHI_F and ISP_XhoI_R (Table S2). The PCR product and plasmid pNZ8037 was digested with FastDigest BamHI and XhoI (Thermo Fisher Scientific), then ligated at 16°C overnight in 3:1 ratio (insert:vector) using T4 DNA ligase (New England BioLabs). After deactivation at 65°C for 10 minutes, the ligation mixture (5 µl) was electroporated into *Lactococcus lactis* subsp. *lactis* NZ9000. Resulting in two strains harboring pNZ8037-*malE-ISP26* (MBP-ISP26) or pNZ8037-*malE* (MBP)*.*

**Purification**

One liter of GM17 (M17 supplemented with 0.4% glucose; Oxoid) containing 10 µg/ml chloramphenicol was inoculated with 10 ml of culture and incubated at 30°C. When the OD_600_ reached approximately 0.5, nisin (N5764; Sigma) was added to 10 ng/ml. Cultures were incubated for another 2 hours after induction, before being harvested by centrifugation (4000g, 30 min, 4°C). Cells were resuspended in 25 ml of column buffer (CB) (NEBExpress® MBP Fusion and Purification System manual; New England BioLabs) and lysed by three passes through a French pressure cell (Aminco; FA-073) at 15 000 PSI. Intact cells and cell debris were removed by centrifugation (20000g, 40 min, 4°C). Gravity flow columns were prepared with 2 ml of amylose resin (E8021; New England BioLabs) that was washed with 10 ml of CB prior to use. The clarified lysate was applied to the column, and the resin was washed with 10 ml of CB. The sample was eluted with 10 ml of CB containing 10 mM maltose in 1 ml fractions. Fractions 2-3 were pooled and concentrated to 100 µl using an Amicon Ultracel-30K centrifugal filter unit (UFC5030; Millipore). Samples were analyzed by SDS-PAGE using Mini-PROTEAN TGX Stain-Free Precast Gels (Bio-Rad).

**Supplementary Results**

**Heterologous expression and purification of hybrid peptides**

To establish a bacterial expression system for the hybrid peptides, *E. coli* BL21(DE3) were initially transformed with pET-3a plasmids, each containing a peptide-encoding gene downstream of an IPTG-inducible promoter. An attempt was made to demonstrate antimicrobial activity directly in culture lysates of transformants encoding the active hybrids (Table 2). However, growth of clones was severely affected both with and without IPTG, indicating a toxic effect of the peptide on *E. coli*. Despite attempts to optimize the inducer concentrations (0.05 to 3 mM IPTG) and harvesting time points post induction (0.5-24 h), no activity could be recovered.

Maltose-binding protein (MBP) is known to solubilize fused proteins and would be expected to detoxify bacteriocin peptides [1–3]. To test this, we fused ISP26 and ISP29 to the C-terminal end of MBP in pMAL-c6T with an in-frame TEV cleavage tag. The TEV cleavage site is reported to be tolerant to a methionine in the P1’ position [4]. For this reason, the TEV cleavage tag was fused to ISP26 such that cleavage would leave no additional N-terminal residues that could interfere with antimicrobial activity. Interestingly, clones harboring this plasmid also exhibited severely attenuated growth only reaching an OD_600_ ~ 0.2-0.3 (compared to OD_600_ > 2 for the control) even without the addition of IPTG. This was observed for all *E. coli* strains tested; DH5α, BL21(DE3) pLysS, and C41(DE3). Cultures would occasionally reach a density comparable to controls, however, these cultures were found to be dominated by disruption mutants of the construct (revealed by Sanger sequencing of the plasmids), suggesting that the ISP26 and ISP29 peptides are toxic in these fusion constructs in *E. coli*.

The native producers of all bacteriocins in the library are Gram-positive species. Therefore, the NICE expression system established in the Gram-positive *Lac. lactis* was attempted next; the MBP-ISP26 recombinant fusion protein was cloned into pNZ8037 downstream of the nisin-inducible promoter (P_nis_) and transformed into *Lac. lactis* NZ9000. The resulting strain exhibited somewhat reduced growth upon nisin induction (10 ng/ml), however, the generation time was only reduced approximately two-fold. The fusion protein was successfully purified from the lysate of this strain (see Figure S2). However, subsequent cleavage of the fusion protein using TEV protease did not yield active bacteriocin peptide, nor was any cleavage of the fusion protein apparent by SDS-PAGE. This could suggest that the compact globular fold of the peptide hinders access to the cleavage site by the protease, or alternatively that the cleavage site is buried in the protein core because of the hydrophobic character of the peptide. However, attempts at performing the TEV cleavage in the presence of guanidine HCl (3 M), urea (2 M) and SDS (0.5%) also failed. We also tested the activity of the purified fusion against *Lac. lactis* IL1403, however, no antimicrobial activity could be observed.

**Supplementary Tables**

**Table S1** Bacteriocins used to create the library of hybrid peptides. Each bacteriocin was split at the position indicated by a space resulting in an N- and C- terminal part, both parts were combined in all combinations to produce a library of hybrid peptides shown in Table 1. Acidic residues (E/D) are underlined

|  | **ISP** | **N-terminal part** |  | **C-terminal part** | **Reference**^†^ |
| --- | --- | --- | --- | --- | --- |
| LliBU* | ISP1 | MWGRILGTVAKYGPKAVSWAWQHK |  | WFLLNMGDLAFRYIQRIWG | Lozo et al. [5] |
| BHTB | ISP8 | MWGRILAFVAKYGTKAVQWAWKNK |  | WFLLSLGEAVFDYIRSIWGG | Hyink et al. [6] |
| AurA53 | ISP15 | MSWLNFLKYIAKYGKKAVSAAWKYK |  | GKVLEWLNVGPTLEWVWQKLKKIAGL | Netz et al. [7] |
| K411 | ISP22 | MAGFLKVVKAVAKYGSKAVKWCWDNK |  | GKILEWLNIGMAVDWIVEQVRKIVGA | Tymo. et al. [8] |
| LacQ | ISP29 | MAGFLKVVQLLAKYGSKAVQWAWANK |  | GKILDWLNAGQAIDWVVSKIKQILGIK | Fujita et al. [9] |
| EpiNI01 | ISP36 | MAAFMKLIQFLATKGQKYVSLAWKHK |  | GTILKWINAGQSFEWIYKQIKKLWA | San. & Upt. et al. [10] |
| SalC | ISP43 | MSALAKLIAKFGYKK |  | IMQLIGEGWTVNQIEKMFK | Tymo. et al. [8] |

* The LliBU sequence used in this study differs from the published sequence in two positions (E26F and I28L).

† Tymo.; Tymoszewska. San. & Upt.; Sandiford & Upton.

**Table S2** Oligonucleotides

| **Primer** | **Sequence (3’-5’)** |
| --- | --- |
| HiFi_ISP26_F | AGAACCTGTACTTCCAGATGGCCGGTTTTCTGAAAGTG |
| HiFI_ISP26_R | AGCTTATTTAATTACCTGCATTATTTGATCCCTAAAATCTGC |
| ISP_BamHI_F | AAAGGATCCGTTTAGGTGTTTTCACGAGC |
| ISP_XhoI_R | AAACTCGAGACGAAAGGCCCAGTCTTTCG |

**Supplementary Figures**

**
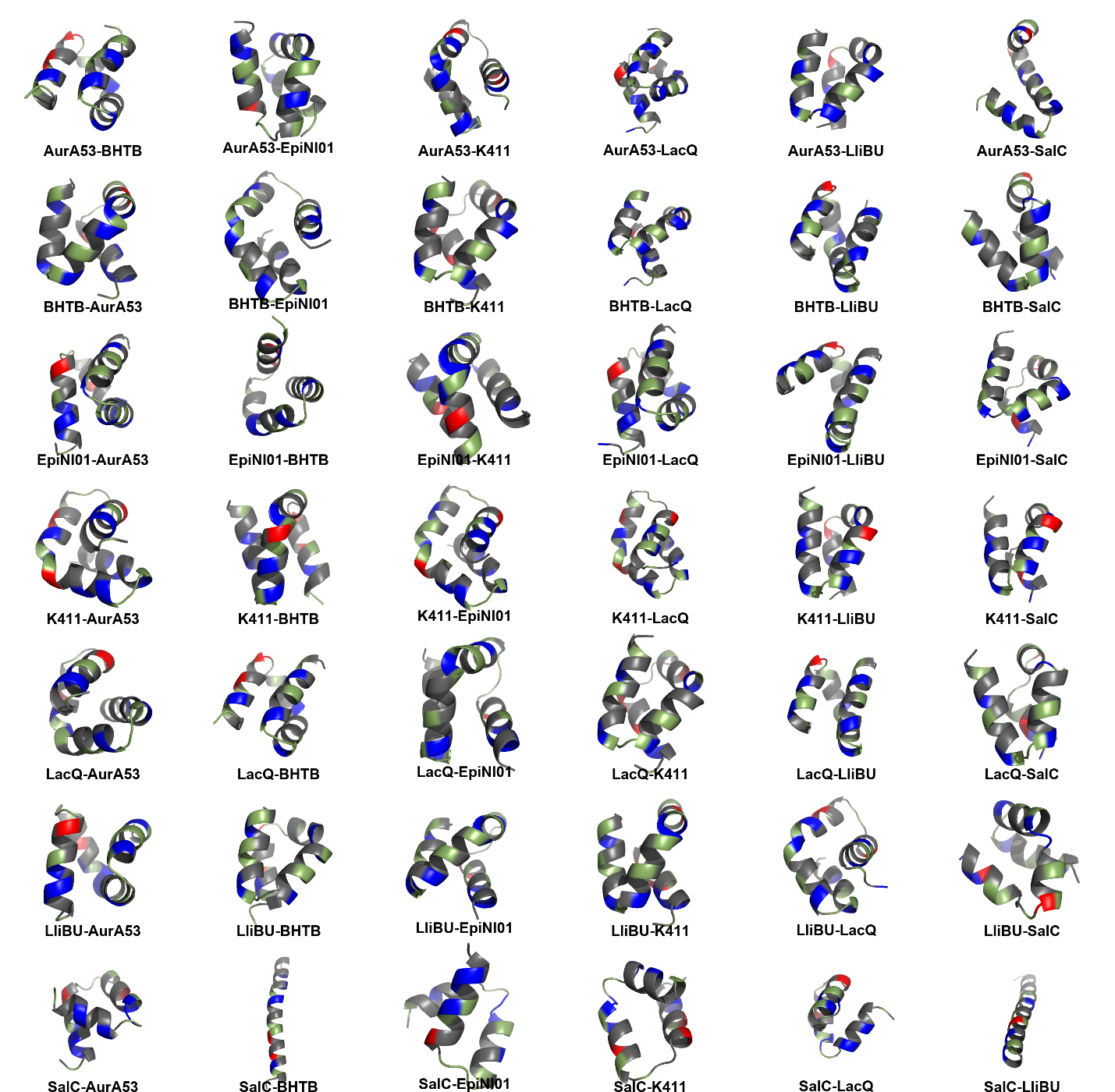
**

**Fig. S1** AlphaFold2 structure predictions of all hybrid peptides in the library. Positively charged amino acids (K, L, H) are colored blue, acidic residues (D, E) are shown in red and hydrophobic residues (F, I, L, M, V, W, A, P) are colored in gray. The image was generated with a script utilizing the PyMOL API and the Python imaging library Pillow [11, 12]

**
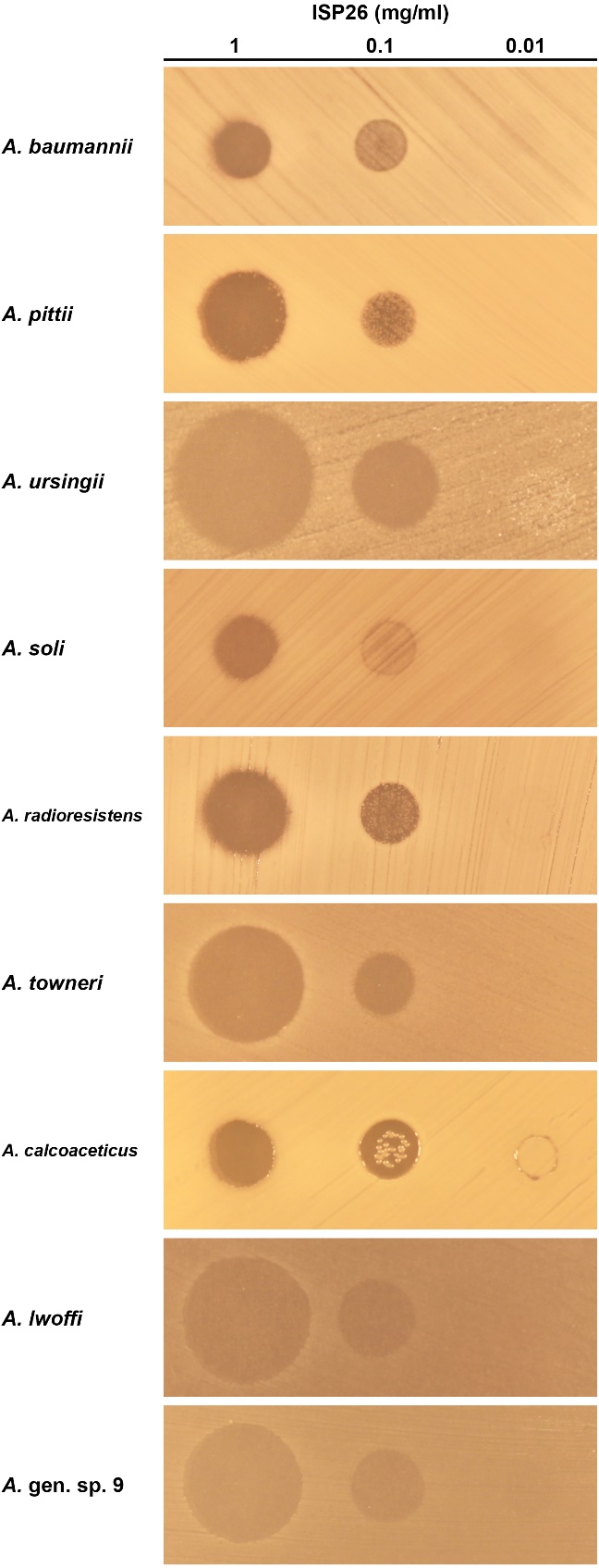
**

**Fig. S2** Spot-on-lawn assay of ISP26 against a selection of *Acinetobacter* species. Synthetically obtained ISP26 was spotted (5 µl) at the indicated concentration. Plates were prepared according to the EUCAST disk diffusion methodology

**
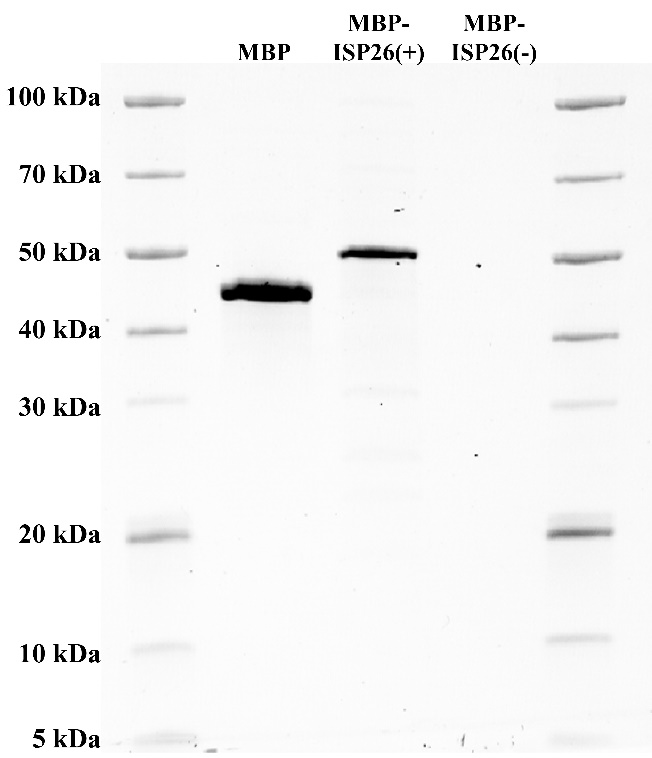
**

**Fig. S3** SDS-PAGE of purified MBP and MBP-ISP26 fusions from *Lac. lactis* NZ9000 (see Supplementary methods). Lane MBP: MBP was purified from a control strain expressing the unchanged *malE* gene supplied in the pMAL-c6T vector (calculated mass of 45.5 kDa). Lane MBP-ISP26(+): Purification of the MBP-ISP26 fusion protein (49.6 kDa), purified from a culture induced (+) with 10 ng/ml nisin. Lane MBP-ISP26(-): Purification of MBP-ISP29 from an uninduced (-) culture

**References**

1. Mesa-Pereira B, Rea MC, Cotter PD, et al (2018) Heterologous Expression of Biopreservative Bacteriocins With a View to Low Cost Production. Frontiers in Microbiology 9:. https://doi.org/10.3389/fmicb.2018.01654

2. Rodrı́guez JM, Martı́nez MI, Horn N, Dodd HM (2003) Heterologous production of bacteriocins by lactic acid bacteria. International Journal of Food Microbiology 80:101–116. https://doi.org/10.1016/S0168-1605(02)00153-8

3. Sachdev D, Chirgwin JM (1998) Solubility of Proteins Isolated from Inclusion Bodies Is Enhanced by Fusion to Maltose-Binding Protein or Thioredoxin. Protein Expression and Purification 12:122–132. https://doi.org/10.1006/prep.1997.0826

4. Kapust RB, Tözsér J, Copeland TD, Waugh DS (2002) The P1′ specificity of tobacco etch virus protease. Biochem Biophys Res Commun 294:949–955. https://doi.org/10.1016/S0006-291X(02)00574-0

5. Lozo J, Mirkovic N, O’Connor PM, et al (2017) Lactolisterin BU, a Novel Class II Broad-Spectrum Bacteriocin from *Lactococcus lactis* subsp. *lactis* bv. diacetylactis BGBU1-4. Appl Environ Microbiol 83:e01519-17. https://doi.org/10.1128/AEM.01519-17

6. Hyink O, Balakrishnan M, Tagg JR (2005) *Streptococcus rattus* strain BHT produces both a class I two-component lantibiotic and a class II bacteriocin. FEMS Microbiol Lett 252:235–241. https://doi.org/10.1016/j.femsle.2005.09.003

7. Netz DJA, Pohl R, Beck-Sickinger AG, et al (2002) Biochemical Characterisation and Genetic Analysis of Aureocin A53, a New, Atypical Bacteriocin from *Staphylococcus aureus*. J Mol Biol 319:745–756. https://doi.org/10.1016/S0022-2836(02)00368-6

8. Tymoszewska A, Ovchinnikov KV, Diep DB, et al (2021) *Lactococcus lactis* Resistance to Aureocin A53- and Enterocin L50-Like Bacteriocins and Membrane-Targeting Peptide Antibiotics Relies on the YsaCB-KinG-LlrG Four-Component System. Antimicrob Agents Chemother 65:. https://doi.org/10.1128/aac.00921-21

9. Fujita K, Ichimasa S, Zendo T, et al (2007) Structural Analysis and Characterization of Lacticin Q, a Novel Bacteriocin Belonging to a New Family of Unmodified Bacteriocins of Gram-Positive Bacteria. Appl Environ Microbiol 73:2871–2877. https://doi.org/10.1128/AEM.02286-06

10. Sandiford S, Upton M (2012) Identification, Characterization, and Recombinant Expression of Epidermicin NI01, a Novel Unmodified Bacteriocin Produced by *Staphylococcus epidermidis* That Displays Potent Activity against Staphylococci. Antimicrob Agents Chemother 56:1539–1547. https://doi.org/10.1128/aac.05397-11

11. Schrödinger, LLC. (2021) The PyMOL Molecular Graphics System, v2.5.2

12. Murray A, Kemenade H van, wiredfool, et al (2024) python-pillow/Pillow: 10.2.0
